# Malaria research in the Central African Republic from 1987 to 2020: an overview

**DOI:** 10.1186/s41182-022-00446-z

**Published:** 2022-09-21

**Authors:** Romaric Nzoumbou-Boko, Guillaume Velut, Romeo-Karl Imboumy-Limoukou, Alexandre Manirakiza, Jean-Bernard Lekana-Douki

**Affiliations:** 1 Laboratoire de Parasitologie, Institut Pasteur de Bangui, PO Box 923, Bangui, Central African Republic; 2 French Military Health Service, French Armed Forces Centre for Epidemiology and Public Health (CESPA), Marseille, France; 3 Unité Évolution, Épidémiologie Et Résistances Parasitaires (UNEEREP), Centre International de Recherche Médicale de Franceville (CIRMF), BP769 Franceville, Gabon; 4 Service d’épidémiologie, Institut Pasteur de Bangui, PO Box 923, Bangui, Central African Republic; 5 Département de Parasitologie-Mycologie Médecine Tropicale, Faculté de Médecine, Université des Sciences de la Santé, Libreville, B.P. 4009 Franceville, Gabon

**Keywords:** Malaria research, Control policy, Database, Publication output, Central African Republic

## Abstract

**Background:**

The national malaria control policy in the Central African Republic (CAR) promotes basic, clinical, and operational research on malaria in collaboration with national and international research institutions. Preparatory work for the elaboration of National Strategic Plans for the implementation of the national malaria control policy includes developing the research component, thus requiring an overview of national malaria research. Here, this survey aims to provide an inventory of malaria research as a baseline for guiding researchers and health authorities in choosing the future avenues of research.

**Methods:**

Data sources and search strategy were defined to query the online Medline/PubMed database using the “medical subject headings” tool. Eligibility and study inclusion criteria were applied to the selected articles, which were classified based on year, research institute affiliations, and research topic.

**Results:**

A total of 118 articles were retrieved and 51 articles were ultimately chosen for the bibliometric analysis. The number of publications on malaria has increased over time from 1987 to 2020. These articles were published in 32 different journals, the most represented being the *Malaria Journal* (13.73%) and the *American Journal of Tropical Medicine and Hygiene* (11.76%). The leading research topics were drug evaluation (52.94%), expatriate patients (23.54%), malaria in children (17.65%), morbidity (13.7%), and malaria during pregnancy (11.76%). The publications’ authors were mainly affiliated with the Institut Pasteur of Bangui (41%), the French Military Medical Service (15.5%), and the University of Bangui (11.7%). Collaborations were mostly established with France, the UK, and the USA; some collaborations involved Switzerland, Austria, Pakistan, Japan, Sri Lanka, Benin, Cameroun, Ivory Coast, and Madagascar. The main sources of research funding were French agencies (28.6%) and international agencies (18.3%). Most studies included were not representative of the whole country. The CAR has the capacity to carry out research on malaria and to ensure the necessary collaborations.

**Conclusion:**

Malaria research activities in the CAR seem to reflect the priorities of national policy. One remaining challenge is to develop a more representative approach to better characterize malaria cases across the country. Finally, future research and control measures need to integrate the effect of COVID-19.

## Background

Malaria remains a major public health problem; this disease is widespread in tropical regions despite increased funding for control programs and major advancements in diagnosis and treatment. Although the prevalence of malaria has decreased recently in parts of Africa, it continues to increase in the Central African Republic (CAR): prevalence increased from 68.9% to 74.71% between 2015 and 2020 [1]. Over the last 20 years, many initiatives have been launched in the CAR, particularly between 2000 and 2013—a period of intensification of malaria control actions—when the CAR benefited from some World Health Organization (WHO) programs and those of other international non-governmental organizations. Initiatives taken in the CAR include the introduction of artemisinin-based combination therapies as the first-line treatment for unconfirmed malaria in 2005, the establishment of a network of community health workers (CHWs) in 2008, the introduction of a rapid diagnostic test (RDT) in 2009, the distribution of long-lasting insecticide-treated nets (LLINs) in 2010, and home-based management of malaria (PECADOM) starting in 2013 [2, 3].

The WHO’s Global Technical Strategy for malaria (GTS) 2016–2030 produced the High Burden to High Impact country-led approach whose actions are tailored to local data and information [4]. Thus, in 2016, the CAR set up a national malaria control policy derived from the GTS; this policy emphasizes policy support through catalyzing innovation and developing malaria research. In addition, this national policy strives to promote basic, clinical, and operational research on malaria at all levels of the health system in collaboration with national and international research institutions. This policy will be implemented through National Strategic Plans (NSP) [5]. The preparatory work for the elaboration of the NSP for malaria surveillance and the development of malaria scientific research in CAR requires an overview of national malaria research.

The challenges for malaria research are numerous in the CAR. The priority and the most pressing issues involve vector control. For example, a study in Bangui showed that mosquitoes can modify their behavior to blood-feed at places and times when humans are not protected, i.e., primarily outside the home environment [6]. In addition, there is a risk that *Anopheles stephensi*—already present in Sudan—becomes established in the CAR; this vector frequents urban areas. However, knowledge on malaria vectors remains limited in the CAR, thereby hampering disease control [7]. Another important issue is insecticide or drug resistance. A recent study showed multiple insecticides resistance in important malaria vectors (*An. gambiae* s.l. and *An. funestus* s.l.) in Bangui [8, 9]. Likewise, there is a multiplicity of factors that may favor the introduction, the emergence and the selection of drug-resistant malaria strains, particularly those resistant to artemisinin derivatives. These factors include the presence of troops from Cambodia, Thailand, Bangladesh, Bhutan and Nepal for the past 10 years in the CAR; all of these countries are located in the area where artemisinin resistance has emerged or neighbor this epicenter of emergence, thus leading to the potential spread of multidrug-resistant *P. falciparum*. Finally, the reservoir of asymptomatic carriage has never been assessed and is therefore an important issue to address to evaluate the potential of residual malaria transmission.

Here, we provide an inventory of malaria research as a baseline to help researchers and health authorities define the avenues for future malaria research in the CAR. This study establishes a database defining the current state of art with a list of the main articles on malaria in a country that has very limited internet connections and all but lacks medical research documentation centers or up-to-date libraries. Research results are commonly communicated in the form of articles published in journals indexed in international bibliographic databases of which the most recognized in biomedical sciences is “Medline” with its electronic interface “PubMed” [10]. This literature review provides a bibliometric profile of the publications in Medline/PubMed on malaria in the CAR over a period of 33 years (from 1987 to 2020).

## Methods

### Data source and search strategy

An online Medline/PubMed database search was conducted from 1 November to 31 January 2021 (last update). The Medline/PubMed database is based on the US National Library of Medicine® (NLM) bibliographic database, which holds more than 28 million references to journal articles in life sciences, published from 1966 by more than 5,200 journals worldwide [11]. The medical subject headings (MESH) tool was used as follows: (((malaria) OR plasmodium) OR anopheles) AND ("Central African Republic" OR "Bangui"))). The retrieved references were then analyzed for the following keywords: malaria, mosquito, antimalarial drug, clinical malaria.

### Eligibility, study inclusion criteria and analysis plan

Retrieved publications were screened to select the reports of primary original studies conducted between 1966 and 2020 exclusively in the CAR. English and French language publications were included. These publications had to refer specifically to human malaria. Articles involving other regions, other pathologies, or animal malaria were excluded. Two authors (R.N-B and G.V) assessed all studies and agreed on those that met the criteria and should therefore be included. Data collected included the number of articles published annually, journal, author affiliations, funding, city of study, types of research (basic, clinical, epidemiological research and health policy and systems research) and research topics. A data extraction form was designed to compile the information available in the publications. Two authors (R.N-B and G.V) independently extracted data from the studies using this form. Any discrepancies in data extraction by the two authors were reconciled through discussion. The data obtained were encoded and processed in Microsoft Excel 2010.

## Results

### Overview of selected articles

**Fig. 1:**
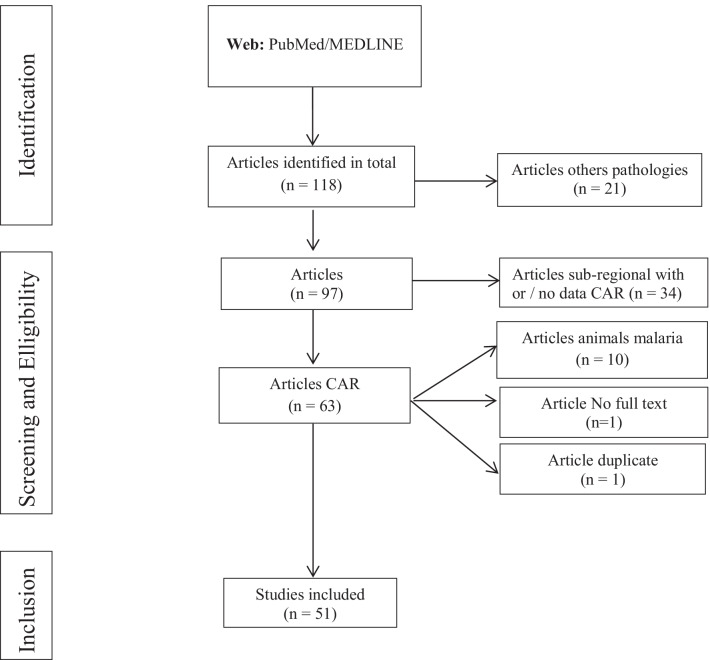
Flowchart of study selection process and search strategy

A total of 118 articles were identified and recorded in the Medline/PubMed database. We discarded 21 articles investigating other pathologies, 34 articles about malaria in the global subregion of central Africa—with or without data from the CAR—10 articles on animal malaria, 1 article with no full text available and 1 article that was a duplicate study. Ultimately, 51 articles were included in our assessment of research on malaria in the CAR (Fig. 1). Most of the retrieved articles were written in English (39/51).

### Trends in malaria research and journals

The first report on malaria in the CAR being published in 1987, Fig. 2 shows the increase in the number of articles published each year. Malaria research in the CAR can be grouped into three phases: 2005–2006 with 3–4 papers per year; 2015–2017 with 4–5 papers per year and 2017 with 4 papers per year. Thus, in 33 years of malaria research in the CAR, there were on average 1.55 articles per year. Over the last 5 years, there has been an increase, with an average of 3.6 articles per year.

**Fig. 2:**
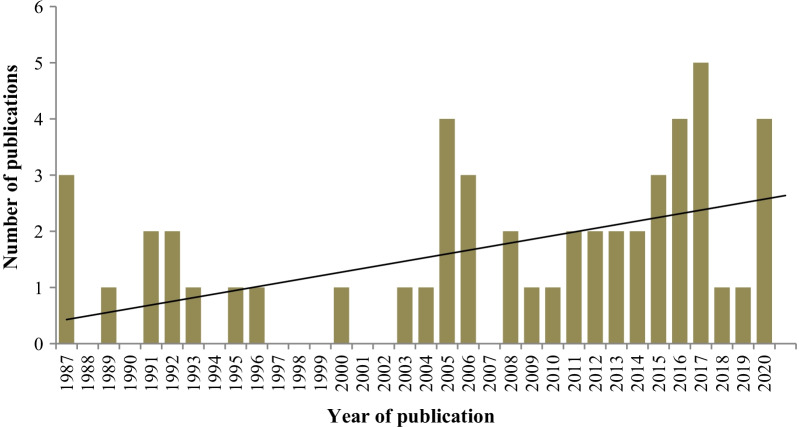
Trend of malaria publications in Central African Republic of 1987 at 2020

These articles were published in 32 different journals, the leading journals being the following: *Malaria Journal* (13.73%), *American Journal of Tropical Medicine and Hygiene* (11.76%), *Bulletin de la Societe de Pathologie Exotique* (7.84%), *Médecine Tropicale* (5.88%) and *Transactions of the Royal Society of Tropical Medicine and Hygiene* (5.88%) (Table 1).

**Table 1:** Journals publishing malaria research from Central African Republic

No.	Journals	Number of articles
1	Malaria Journal	7
2	The American Journal of Tropical Medicine and Hygiene	6
3	Bulletin de la Société de Pathologie Exotique	4
4	Médecine Tropicale: Revue du Corps de Sante Colonial	3
5	Transactions of the Royal Society of Tropical Medicine and Hygiene	3
6	Parasites and Vectors	2
7	Trials	1
8	Parasite	1
9	Médecine et Sante Tropicales	1
10	Médecine et Maladies Infectieuses	1
11	Malaria Research and Treatment	1
12	The Journal of the Japanese Association for Infectious Diseases	1
13	Journal of Tropical Medicine	1
14	Journal of Hygiene, Epidemiology, Microbiology, and Immunology	1
15	International Journal of Technology Assessment in Health Care	1
16	Emerging Infectious Diseases	1
17	East African Journal of Public Health	1
18	Cahiers Santé	1
19	BMC research notes	1
20	BMC public health	1
21	BMC military medical research	1
22	BMC infectious diseases	1
23	Biomedicine & Pharmacotherapy	1
24	Antimicrobial Agents and Chemotherapy	1
25	Annales de Pédiatrie	1
26	Annales de Biologie Clinique	1
27	American Journal of Epidemiology	1
28	The Journal of Public Health in Africa	1
29	Pathogens and Global Health	1
30	Cureus	1
31	Interdisciplinary Perspectives on Infectious Diseases	1
32	Acta Tropica	1

### Research topics addressed in the selected articles

Most publications involved clinical research (41.7%). Epidemiological and basic research accounted for 23.52% and 21.56% of the articles, respectively. Health policy and systems research was less represented (13.22%).

**Fig. 3:**
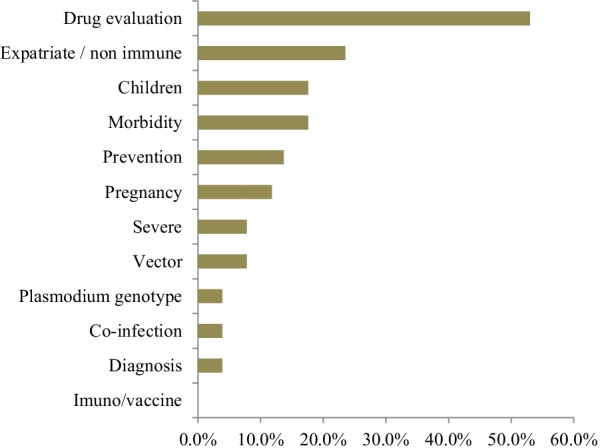
Areas of focus for malaria research in Central African Republic

Regarding the research topics, drug evaluation (efficacy and resistance) was the most common topic of investigation with 27 publications (52.94%) (Fig. 3). Publications on expatriate patients (23.54%), children (17.65%), and morbidity (17.65%) were followed by papers on prevention (13.72%) and malaria during pregnancy (11.76%). Less frequently addressed topics included severe malaria cases (7.84%), vector control (7.84%), diagnosis (3.9%), *Plasmodium* genotype (3.9%), and co-infection (3.9%) (Table 2). There were no studies on immunology or vaccine development in the selected articles.

**Table 2:** Summary of the studies included in the review

No.	Authors	Publication year	Field of malaria research	Location or district
1	Nzoumbou-Boko R et al. [12]	2020	Co-infection/morbidity	Bangui, Boali, Bossembelé, Pissa
2	Malik JA et al. [13]	2020	Drug resistance/prevention/expatriate	Kaga-Bandoro
3	Nzoumbou-Boko R et al. [14]	2020	Drug resistance	Bangui
4	Ferdinand DY et al. [15]	2020	Morbidity	Paoua and Carnot
5	Guerra RI et al. [16]	2019	Prevention/expatriate	Bangui
6	Kamgang B et al. [15]	2018	Vector	Bangui
**7**	Manirakiza A et al. [16]	2017	Pregnant woman	Bangui
8	Ruckstuhl L. et al. [19]	2017	Morbidity	Paoua/Marcounda
9	Bichara C. et al. [20]	2017	Parasite genotype/children/severe	C.A.R
10	Fernando, S.D et al. [21]	2017	Prevention/expatriate	Bangui
11	Ole Sangba, M.L. et al. [8]	2017	Vector	Bangui
12	Ndiath, M.O et al. [22]	2016	Vector	Bangui
13	Sangba, M.L.O et al. [9]	2016	Vector	Bangui
14	Javelle E. et al. [23]	2016	Drug resistance/expatriate	C.A.R
15	Creach, M.D et al. [24]	2016	Prevention/expatriate	C.A.R
16	Bobossi-Serengbe G. et *al*. [25],	2015	Drug/children/severe	Bangui
17	Madamet M. et al. [26]	2015	Drug resistance/expatriate/severe	Bangui
18	Serengbe, G.B et al. [27]	2015	Prevention	Lobaye, Ouham, Ouaka Bangui
19	Djalle D. et al. [28]	2014	Diagnostics	Bangui
20	Djalle D. et al. [29]	2014	Drug /children	Bangui
21	Nambei W. S et al. [30]	2013	Drug resistance/children	Bangui
22	Manirakiza A. et al. [31]	2013	Drug/ pregnant women/co-infection	Bangui
23	Danguy des D. M. et al. [32]	2012	Drug	C.A.R
24	Manirakiza A et al. [33]	2012	Diagnostics/ pregnant woman	Bangui
25	Manirakiza A. et al. [34]	2011	Morbidity/ pregnant woman	Bangui
26	Manirakiza, A. et al. [35]	2011	Drug/ pregnant woman	Bangui
27	Gresenguet G. et al. [36]	2010	Drug	C.A.R
28	Manirakiza M. et al. [37]	2009	Drug	Bangui
29	Dolmazon V. et al. [38]	2008	*Plasmodium* genotype	Bangui
30	Nambei W.S et al. [39]	2008	Drug/children	Bangui
31	Matsika-Claquin M. et al. [40]	2006	Drug / expatriate/prevention	Bangui
32	Menard D. et al. [41]	2006	Drug resistance	Bangui
33	Menard.D et al. [42]	2006	Drug resistance	Bangui
34	Menard.D et al. [43]	2005	Drug/expatriate	Bangui
35	Menard.D et al. [44]	2005	Drug/children	Bangui
36	Menard.D et al. [45]	2005	Drug resistance	Bangui
37	Nambei W. S et al. [46]	2005	Drug resistance	Bangui
38	Bobossi Serengbe G. et *al.* [47]	2004	Children/severe	Bangui
39	Bergeri I. et al. [48]	2003	Drug/children	Bambari, Bangassou, Bangui, Bossangoa, Berberati
40	Rowe A. K et al. [49]	2000	Children	C.A.R
41	Baron E. et al. [50]	1996	Morbidity/expatriate	Bangui, Bouar
42	Miyashita N. et al. [51]	1995	Drug/prophylaxis	No precision
43	Garin D. et al. [52]	1993	Drug/expatriate/prevention	Bouar
44	Lanckriet C. et al. [53]	1992	Morbidity/children	Bangui
45	Delmont, J. et al. [54]	1992	Drug resistance	seven towns in C.A.R
46	Darie H. et al. [55]	1991	Morbidity/expatriate	C.A.R
47	Testa J. et al. [56]	1991	Morbidity/expatriate	Bangui
48	Belec L et al. [57]	1989	Drug resistance/expatriate	Berberati
49	Pierce P. F et al. [58]	1987	Drug resistance	No Bangui
50	Testa J. et al. [59]	1987	Pregnant woman /morbidity	Bangui
51	Delmont J. et al. [60]	1987	Drug resistance/ children	Bangui

### Affiliations and funding

First authors were affiliated with CAR institutions and French institutions in 32 (62.74%) and 9 (17.64%) publications, respectively. The other authoring institutions were located in the UK, the USA, Switzerland, Pakistan, Japan, Sri Lanka, and Ivory Coast. Last authors were affiliated with CAR and French institutions in 17 (38.6%) publications each, and the other last authors’ institutions were located in the USA, Switzerland, Cameroun, Japan, Sri Lanka, and Madagascar. The reported studies were mainly carried out at the Institut Pasteur of Bangui (41%), the University of Bangui (11.7%), French Military Medical Services (15.5%), and the CAR Ministry of Public Health (5.8%). Other co-authoring institutions were Aix-Marseille University, Bangui National Hospital, Bouake Regional Hospital, Georgetown University Hospital, Center of Disease Control and Prevention in Atlanta (USA), and the University of Colombo. Twenty-seven (53%) of these studies were carried out in collaboration between several institutes and research centers; among them, 12 involved more than two institutions (28.6%). Funding was acknowledged in only 28 publications (55%), of which 9 benefited from cofunding (21.4%). The main sources of these funds were French agencies (28.6%) and international agencies (18.3%).

## Discussion

The bibliometric analysis focused on a review of studies to assess the type and amount of malaria research conducted in CAR from 1987 to 2020. The publication of the first paper on malaria in the CAR coincided with the spread of chloroquine resistance in Africa in the 1980s, and the three articles of this period addressed chloroquine resistance. Similarly, in other areas of central Africa (i.e., the Democratic Republic of Congo, the Republic of Congo, and Cameroon), numerous publications on chloroquine resistance have been published [61–63]. The annual number of CAR malaria articles published varied from year to year and has been increasing since 1987. The introduction of artemisinin in the treatment regimen beginning in the 2000s led to assessments of its efficacy and of other antimalarial drugs, explaining the number of articles published between 2005 and 2006 involving the assessment of antimalarial drugs. From 2015 to 2017, there was an average of four articles per year, corresponding to the publication of the first articles on the malaria vectors and the concurrent deployment of soldiers for peacekeeping missions in the CAR during the periods of unrest in 2013, and ending with the publications on imported malaria cases in 2017. This study showed that the top two journals, *Malaria Journal* and the *American Journal of Tropical Medicine and Hygiene* were the same as those reported in a bibliometric analysis of malaria research in Malawi from 1984 to 2016 [64]. A bibliometric analysis of the literature on malaria vector resistance from 1996 to 2015 showed that *Malaria Journal* was the leading journal, although it was established only in 2002 [65].

Almost all the studies reported in the articles were derived from primary research, accounting for 50 out of 51 papers (98%). The types of malaria research conducted in the CAR were exactly the same types of malaria research carried out in Malawi, with mostly clinical research and few health policy and systems research studies [64]. Clinical research has been revealed in bibliometric analysis of research on meningitis and other infectious diseases as the leading type of approach and topic of research [66]. The research topics and areas of focus for malaria research in the CAR matched the priorities of the topics listed in the national policy and the ground situation in the country, namely drug evaluation, and malaria in children and in pregnant women. However, efforts must be made in the field of vector control and diagnosis, which are the pillars of any control strategy, in particular regarding parasite genotype, immunological profile, and vaccine development. Drug evaluation was also the leading research topic in a study carried out in the Greater Mekong subregion [67].

Another important aspect revealed in this bibliometric analysis was the level of collaboration. Based on a review of publications, our survey revealed a high rate of collaboration between a CAR institution and one or more foreign/international institutions. Research institutes on almost every continent have contributed to the study of malaria in the CAR and this multiple and diversified collaboration strengthens the local capacity for malaria research and documents the local malaria situation. International funding was acknowledged in more than half of the articles selected and no local funding was cited. In contrast, in a study on the investment in malaria research in sub-Saharan Africa, Tanzania, Uganda, Kenya, Malawi, and Ghana are all countries showing greater funding, with the CAR being ranked among countries with no funds allocated for research [68]. This lack of investment may also explain the low scientific production regarding malaria research in the CAR compared with other endemic countries. Furthermore, the limited resources and expertise in the CAR means that the country depends on international collaboration to ultimately control the fatal infectious disease of malaria.

We found that 70% of the studies on malaria in the CAR had been carried out in the capital (Bangui), probably because the city is readily accessible to foreign researchers. However, the representativeness of these studies may be called into question, because Bangui has about 812,407 inhabitants for an area of 67 km^2^, whereas the vast majority of the CAR’s 6,091,097 inhabitants are spread across an area of 623,000 km^2^ [69]. Restricting studies to Bangui can be attributed to the lack of qualified infrastructures in the outlying provinces and especially to conflicts, unrest, and the military crisis, which have a greater impact in the provinces. Notwithstanding, a study carried out in the network of sentinel sites set up for epidemiological surveillance in the CAR revealed spatial variability in malaria prevalence, with high prevalence in rural and semi-urban areas [14]. Nevertheless, this bibliographic survey provides a sound scientific basis for guiding future control measures against malaria in the CAR and allowed domestic experts to identify the needs for research on malaria in this geographically and digitally landlocked country, with no direct access to broadband internet and connectivity issues.

Finally, future research and control measures will require integrating the effect of COVID-19. In terms of control measures, the COVID-19 pandemic has severely affected health systems in general, including the malaria program with decreased access to LLINs and antimalarial drugs, two key malaria control measures, leading to an increase in morbidity (estimated at 5% to < 10%) and an increase in mortality (estimated at 40% to < 60%) in the CAR, in 2020 compared to the pre-COVID-19 period [70]. Furthermore, the COVID-19 pandemic has also affected the progress of malaria research in the CAR in various ways. Clinical malaria research most carried out in the CAR has been discontinued because individuals have stopped attending health facilities out of fear of exposure to COVID-19, or CHWs and other healthcare staff avoid working in close proximity to febrile patients who may have a high risk of COVID-19 infection. The extensive and uncontrolled use of chloroquine, hydroxychloroquine, and artemisinin derivatives—all antimalarial drugs—and azithromycin—an antibiotic with an antimalarial effect—during the COVID-19 pandemic in the CAR may affect the malaria treatment regimen An interventional study with a combination of control strategies involving vector control, vector surveillance and parasite drug resistance monitoring is to be considered primarily for a host–pathogen–vector–environment interaction approach.

## Conclusion

In summary, the increase in the number of malaria-related articles in the CAR is promising, because various aspects of malaria research have been studied. The CAR has a capacity for malaria research and can rely on fruitful collaborations. Unfortunately, most studies had been carried out on the population in Bangui, revealing a lack of representativeness of these studies for the rest of the country. Malaria research activity in the CAR may reflect the priorities of the national policy, but efforts must be made to focus on vector control and diagnosis. This survey provides a sound scientific basis for domestic public health experts to refine and optimize their bibliographic research on malaria. The challenge now lies in developing the representativeness of studies, assessing the immunological profile of the population and the burden of asymptomatic malaria, as well as characterizing the residual reservoir and measuring transmission rates, including anthropological considerations, and developing educational support actions. Future research and control measures need to integrate the effect of COVID-19.

## Data Availability

The database of this study is available from the corresponding author upon request.

## Abbreviations

WHO: World Health Organization
CHWs: Community Health Workers
RDT: Rapid diagnostic test
LLINs: Long-lasting insecticide-treated nets
PECADOM: Home-based management of malaria
GTS: Global Technical Strategy for malaria
NSP: National Strategic Plans
NLM: National Library of Medicine
MESH: Medical subject headings
COVID-19: Coronavirus disease 2019

## References

[1] WHO. World malaria report 2021. Geneva: World Health Organization; 2021.

[2] WHO. Profile Central African Republic. World Health Organization. 2018.

[3] Plan stratégique national de lutte contre le paludisme 2012–2016. Ministère de la santé publique, de la population et de la lutte contre le VIH, RCA. 2012.

[4] OMS. Global Technical Strategy for Malaria 2016–2030. World Health Organization; 2015.p.1–32.

[5] Politique national de lutte contre le paludisme de la RCA, Programme National de lutte contre le paludisme, Ministère de la Santé et de la population, 32 pages, 2016.

[6] Sangbakembi-Ngounou C, Costantini C, Longo-Pendy NM, Ngoagouni C, Akone-Ella O, Rahola N, Ayala D (2022). Diurnal biting of malaria mosquitoes in the Central African Republic indicates residual transmission may be “out of control”. Proc Natl Acad Sci.

[7] Abubakr M, Sami H, Mahdi I, Altahir O, Abdelbagi H, Mohamed NS, Ahmed A (2022). The Phylodynamic and Spread of the Invasive Asian Malaria Vectors, *Anopheles stephensi*, in Sudan. Biology.

[8] Ole Sangba ML, Sidick A, Govoetchan R (2017). Evidence of multiple insecticide resistance mechanisms in *Anopheles gambiae* populations in Bangui, Central African Republic. Parasit Vectors.

[9] Sangba ML, Deketramete T, Wango SP (2016). Insecticide resistance status of the *Anopheles funestus* population in Central African Republic: a challenge in the war. Parasit Vectors.

[10] NIH. MEDLINE Fact Sheet 2017, available: http://wayback.archive-it. org/org 350/20180312141554/https://www.nlm.nih.gov/pubs/factsheets/ medline.html.

[11] MEDLINE: Overview 2022, available: https://www.nlm.nih.gov/medline/ medline_overview.html.

[12] Nzoumbou-Boko R, Panté-Wockama CG, Ngoagoni C, Petiot N, Legrand E, Vickos U, Gody JC, Manirakiza A, Ndoua C, Lombart JP, Ménard D (2020). Molecular assessment of kelch13 non-synonymous mutations in *Plasmodium falciparum* isolates from Central African Republic (2017–2019). Malar J.

[13] Javaria Arshad Malik (2020). Aqib Nadeem, Zahabia Khalid, Yasser Nadeem, Sehrish Zaffar, Amer H Siddiqui. Development of Resistance Against Mefloquine Prophylaxis in Peace-Keeping Forces in the Central African Republic, Cureus.

[14] Nzoumbou-Boko R, Yambiyo BM, Ngoagouni C, Vickos U, Manirakiza A (2020). Malaria in Febrile Patients at Sentinel Sites for Influenza Surveillance in the Central African Republic from 2015 to 2018. Interdiscip Perspect Infect Dis.

[15] Djerandouba YF, Bessimbaye N, Nzalapan S, Bekaka YO, Mbailao R, Ndoua C, Sergio L, Richard A (2020). Evaluation of the effectiveness of community health workers in the fight against malaria in the Central African Republic (2012–2017). Trans R Soc Trop Med Hyg.

[16] Rosio IG, Marianela O, Hugo OV, Danett KB, Mariana R, Christopher NM, Wesley RC (2019). A cluster of the first reported Plasmodium ovale spp infections in Peru occurring among returning UN peace-keepers, a review of epidemiology, prevention and diagnostic challenges in nonendemic regions. Malar J.

[17] Basile K, Williams T, Carine N, Claire S-N, Murielle W, Jacob MR, Charles SW (2018). Exploring insecticide resistance mechanisms in three major malaria vectors from Bangui in Central African Republic. Pathog Glob Health..

[18] Alexandre M, Eugène S, Richard NN, Sandrine M, Samuel GG, Rock MD, Gislain GBB, Jean MM, Jean D, Gérard G, Abdoulaye S (2017). A brief review on features of falciparum malaria during pregnancy. J Public Health Afr..

[19] Ruckstuhl L, Lengeler C, Moyen JM, Garro H, Allan R (2017). Malaria case management by community health workers in the Central African Republic from 2009–2014: overcoming challenges of access and instability due to conflict. Malar J.

[20] Bichara C, Flahaut P, Costa D, Bienvenu AL, Picot S, Gargala G (2017). Cryptic *Plasmodium ovale* concurrent with mixed *Plasmodium falciparum* and *Plasmodium malariae* infection in two children from Central African Republic. Malar J.

[21] Fernando SD, Booso R, Dharmawardena P, Harintheran A, Raviraj K, Rodrigo C, Danansuriya M, Wickremasinghe R (2017). The need for preventive and curative services for malaria when the military is deployed in endemic overseas territories: a case study and lessons learned. Mil Med Res.

[22] Ndiath MO, Eiglmeier K, Olé Sangba ML, Holm I, Kazanji M, Vernick KD (2016). Composition and genetics of malaria vector populations in the Central African Republic. Malar J.

[23] Javelle E, Madamet M, Gaillard T, Velut G, Surcouf C, Michel R (2016). Delayed Onset of *Plasmodium falciparum* Malaria after Doxycycline Prophylaxis in a Soldier Returning from the Central African Republic. Antimicrob Agents Chemother.

[24] Créach MA, Velut G, de Laval F, Briolant S, Aigle L, Marimoutou C, Deparis X, Meynard JB, Pradines B, Simon F, Michel R, Mayet A (2016). Factors associated with malaria chemoprophylaxis compliance among French service members deployed in Central African Republic. Malar J.

[25] Bobossi-Serengbe G, Gody JC, Fioboy R, Elowa JB, Manirakiza A (2015). Comparison of the effectiveness of artemether and quinine for treatment of severe malaria in children, Bangui, Central African Republic. Bull Soc Pathol Exot.

[26] Madamet M, Gaillard T, Velut G, Ficko C, Houzé P, Bylicki C (2015). Malaria Prophylaxis Failure with Doxycycline. Central African Republic, Emerg Infect Dis.

[27] Serengbe GB, Moyen JM, Fioboy R, Beyam EN, Kango C, Bangue C, Manirakiza A (2015). Knowledge and perceptions about malaria in communities in four districts of the Central African Republic. BMC Res Notes.

[28] Djallé D, Gody JC, Moyen JM, Tekpa G, Ipero J, Madji N, Breurec S, Manirakiza A (2014). Performance of Paracheck™-Pf, SD Bioline malaria Ag-Pf and SD Bioline malaria Ag-Pf/pan for diagnosis of falciparum malaria in the Central African Republic. BMC Infect Dis.

[29] Djallé D, Njuimo SP, Manirakiza A, Laganier R, Le Faou A, Rogier C (2014). Efficacy and safety of artemether + lumefantrine, artesunate + sulphamethoxypyrazine-pyrimethamine and artesunate + amodiaquine and sulphadoxine-pyrimethamine + amodiaquine in the treatment of uncomplicated falciparum malaria in Bangui, Central African Republic: a randomized trial. Malar J.

[30] Nambei WS, Lango Yaya E, Pounguinza S, Achonduh O, Bogon A, Lengande R (2013). Efficacy and safety of antimalarial combinations for treatment of uncomplicated malaria in children in Bangui. Central African Republic Med Sante Trop.

[31] Manirakiza A, Sepou A, Serdouma E, Gondje S, Bata GG, Moussa S, Boulay A, Moyen JM, Sakanga O, Le-Fouler L, Kazanji M, Vray M (2013). Effectiveness of two antifolate prophylactic strategies against malaria in HIV-positive pregnant women in Bangui, Central African Republic: study protocol for a randomized controlled trial (MACOMBA). Trials.

[32] Danguy des Déserts M, Montelescaut E, Di Costanzo L, Commandeur D, Nguyen BV, Ould-Ahmed M, Taudon N, Drouillard I. Severe imported Falciparum malaria treated with artesunate. Ann Biol Clin Paris. 2012; 70(6):733–40. 10.1684/abc.2012.0761 23207821

[33] Manirakiza A, Serdouma E, Heredeïbona LS, Djalle D, Madji N, Moyen M (2012). Rational case management of malaria with a rapid diagnostic test, Paracheck Pf®, in antenatal health care in Bangui. Central African Republic BMC Public Health.

[34] Manirakiza A, Soula G, Laganier R, Klement E, Djallé D, Methode M, Madji N, Heredeïbona LS, Le Faou A, Delmont J (2011). Pattern of the Antimalarials Prescription during Pregnancy in Bangui. Central African Republic Malar Res Treat.

[35] Manirakiza A, Serdouma E, Djalle D, Soula G, Laganier R, Madji N, Moyen M, Faou A, Delmont J (2011). Relatively low prevalence of peripheral and placental Plasmodium infection at delivery in Bangui, central african republic. J Trop Med.

[36] Gresenguet G, Moyen M, Koffi B, Bangamingo JP (2010). Policy brief on improving access to artemisinin-based combination therapies for malaria in Central African Republic. Int J Technol Assess Health Care.

[37] Manirakiza A, Njuimo SP, Le Faou A, Malvy D, Millet P (2009). Availability of antimalarial drugs and evaluation of the attitude and practices for the treatment of uncomplicated malaria in Bangui, Central African Republic. East Afr J Public Health.

[38] Dolmazon V, Matsika-Claquin MD, Manirakiza A, Yapou F, Nambot M, Menard D (2008). Genetic diversity and genotype multiplicity of *Plasmodium falciparum* infections in symptomatic individuals living in Bangui (CAR). Acta Trop.

[39] Nambei WS, Doui-Doumgba A, Bobossi G, Siadoua MD, Madji N, Folokete R (2008). Efficacy of artemether in the treatment of uncomplicated *Plasmodium falciparum* malaria in children aged 6–60 months of age in Bangui (Central African Republic). Sante.

[40] Matsika-Claquin MD, Ménard D, Fontanet AL, Ngwhotue A, Sarda J, Talarmin A (2006). Efficacy of chloroquine-proguanil malaria prophylaxis in a non-immune population in Bangui, Central African Republic: a case-control study. Trans R Soc Trop Med Hyg.

[41] Menard D, Yapou F, Manirakiza A, Djalle D, Matsika-Claquin MD, Talarmin A (2006). Polymorphisms in pfcrt, pfmdr1, dhfr genes and *in vitro* responses to antimalarials in *Plasmodium falciparum* isolates from Bangui, Central African Republic. Am J Trop Med Hyg.

[42] Menard D, Djalle D, Yapou F, Manirakiza A, Talarmin A (2006). Frequency distribution of antimalarial drug-resistant alleles among isolates of *Plasmodium falciparum* in Bangui, Central African Republic. Am J Trop Med Hyg.

[43] Menard D, Matsika-Claquin MD, Djalle D, Yapou F, Manirakiza A, Dolmazon V (2005). Association of failures of seven-day courses of artesunate in a non-immune population in Bangui, Central African Republic with decreased sensitivity of *Plasmodium falciparum*. Am J Trop Med Hyg.

[44] Menard D, Djalle D, Manirakiza A, Yapou F, Siadoua V, Sana S (2005). Drug-resistant malaria in Bangui, Central African Republic: an in vitro assessment. Am J Trop Med Hyg.

[45] Menard D, Madji N, Manirakiza A, Djalle D, Koula MR, Talarmin A (2005). Efficacy of chloroquine, amodiaquine, sulfadoxine-pyrimethamine, chloroquine-sulfadoxine-pyrimethamine combination, and amodiaquine-sulfadoxine-pyrimethamine combination in Central African children with noncomplicated malaria. Am J Trop Med Hyg.

[46] Nambei WS, Gbagba FE, Ndémanga J, Doui-Doumgba K, Diallo TO, Ndadjimbaye V, Flori P (2005). Efficacy comparison between anti-malarial drugs in Africans presenting with mild malaria in the Central Republic of Africa: a preliminary study. Parasite.

[47] Bobossi Serengbe G, Ndoyo J, Gaudeuille A, Longo JD, Bezzo ME, Ouilibona SF, Ayivi B (2004). An update on severe pediatric malaria in Central Africa hospital units. Med Mal Infect.

[48] Bergeri I, Zoguéreh DD, Madji N, Barrau K, Delmont J, Namsenmo A (2003). *In vivo* evaluation of chloroquine therapeutic efficacy in uncomplicated *Plasmodium falciparum* malaria in Central African Republic in 1997 and 1998. Bull Soc Pathol Exot.

[49] Rowe AK, Hamel MJ, Flanders WD, Doutizanga R, Ndoyo J, Deming MS (2000). Predictors of correct treatment of children with fever seen at outpatient health facilities in the Central African Republic. Am J Epidemiol.

[50] Baron E (1996). Incidence of malaria in the French Army in Central African Republic from 1988 to 1993. Med Trop.

[51] Miyashita N, Karino T, Nagatomo Y, Yoshida K, Nakajima M, Okimoto N (1995). A case of *Plasmodium ovale* malaria with thrombocytopenia and an abnormality grade in FDP concentration despite the use of chloroquine as a malaria prophylaxis. JAMA.

[52] Garin D, Lamarque D, Ringwald P, Dupuy O, Chaulet JF (1993). Efficacy of chloroquine-proguanil chemoprophylaxis against malaria in the Central African Republic. Trans R Soc Trop Med Hyg.

[53] Lanckriet C, Bureau JJ, Capdevielle H, Gody JC, Olivier T, Siopathis RM (1992). Morbidity and mortality in the pediatric service of Banqui (Central African Republic) during the year 1990. Implications for public health Ann Pediatr (Paris).

[54] Delmont J, Testa J, Courtois P, Capdevielle H, Le Tien C, Roungou JB. Persistence of low levels of Plasmodium falciparum resistance to chloroquine in the autochthonous population of the Central African Republic. J Hyg Epidemiol Microbiol Immunol. 1992;36(4):362–7. 1300352

[55] Darie H, Reyle Y, Hovette P, Touze JE (1991). Current aspects of malaria in expatriates in the Central African Republic. Med Trop (Mars).

[56] Testa J1, Awodabon J, Lagarde N, Olivier T, Delmont J. Interest in placental apposition as an epidemiological marker for malaria Bull Soc Pathol Exot. 1991;84(5 Pt 5):473–9. 1819396

[57] Belec L, Bouree P, Testa J, Delmont J, Quenum B, Georges AJ (1989). Extension of multiple drug resistant *Plasmodium falciparum* malaria in Africa: report of a Central African case. Biomed Pharmacother.

[58] Pierce PF, Milhous WK, Campbell CC (1987). Clinical and laboratory characterization of a chloroquine-resistant *Plasmodium falciparum* strain acquired in the Central African Republic. Am J Trop Med Hyg.

[59] Testa J, Baquillon G, Delmont J, Kamata G, Ngama G (1987). Pregnancy and blood parasite indices of *Plasmodium falciparum* (results of a study in Bangui (Central African Republic)). Med Trop (Mars).

[60] Delmont J, Testa J, Monges P, Limbassa J, Georges AJ, Faugère B. Status of drug resistance of Plasmodium falciparum in the Central African Republic. Results of studies carried out between 1984 and 1986, Bull Soc Pathol Exot Filiales.1987; 80(3 Pt 2):434–42. 3319251

[61] Delacollette C, Cibanguka J, Buregea H, Nkera J (1987). Compared response to chloroquine, fansidar, quinine, and quinidine slow release of infections with *Plasmodium falciparum* in the Kivu region of Zaire. Acta Leiden.

[62] Simon F, Porte J, Verdier F, Guigon D, Drouville C, Le Bras J (1987). Drug sensitivity of malaria in a population of children in Pointe-Noire, Congo, in the first half of 1986. Bull Soc Pathol Exot Filiales.

[63] Hengy C, Garrigue G, Abissègue B, Ghogomu NA, Gazin P, Gelas H, Kouka-Bemba D, Le Bras J, Jambou R. Surveillance of *Plasmodium falciparum* drug sensitivity in Yaounde and its surroundings (Cameroon). *In vivo* and *in vitro* study. Bull Soc Pathol Exot Filiales. 1989; 82(2):217–23. 2663214

[64] Mwendera CA, de Jager C, Longwe H, Hongoro C, Mutero CM, Phiri KS (2017). Malaria research in Malawi from 1984 to 2016: a literature review and bibliometric. Malar J.

[65] Waleed M. Sweileh, Ansam F. Sawalha, Samah W. Al-Jabi, Sa’ed H. Zyoud, Naser Y. Shraim and Adham S. Abu-Taha. A bibliometric analysis of literature on malaria vector resistance: (1996 – 2015). Globalization and Health (2016) 12:76. 10.1186/s12992-016-0214-4. 10.1186/s12992-016-0214-4 PMC5123357 27884199

[66] Miguel BR, Frederico ACF, Manoel JT, Eberval GF (2021). The Most Influential Papers in Infectious Meningitis Research: A Bibliometric Study. Neurol India.

[67] Walter R. Malaria research in the Greater Mekong Subregion: an overview. Southeast Asian J Trop Med Public Health. 2013; 44 Suppl 1:231–48; discussion 306–7. 24159834

[68] Head MG, Goss S, Gelister Y, Alegana V, Brown RJ, Clarke SC, Fitchett JRA, Atun R, Scott JAG, Newell ML, Padmadas SS, Tatem AJ (2017). Global funding trends for malaria research in sub-Saharan Africa: a systematic analysis. Lancet Glob Health.

[69] Cartographie numérique du RGPH-4, ICASEES, 2021.

[70] Weiss DJ, Bertozzi-Villa A, Rumisha SF, Amratia P, Arambepola R, Battle KE (2021). Indirect effects of the COVID-19 pandemic on malaria intervention coverage, morbidity, and mortality in Africa: a geospatial modelling analysis. Lancet Infect Dis.

